# 新污染物诱导12种细胞核受体相关活性的机器学习预测模型

**DOI:** 10.3724/SP.J.1123.2024.12008

**Published:** 2025-08-08

**Authors:** Jianqing LI, Tianqin WANG, Yuefa TENG, Lei GUO, Yang HUANG, Fei LI

**Affiliations:** 1.鲁东大学化学与材料科学学院，山东 烟台 264025; 1. College of Chemistry and Material Science，Ludong University，Yantai 264025，China; 2.中国科学院海岸带环境过程与生态修复重点实验室（烟台海岸带研究所），山东省海岸带环境过程重点实验室，中国科学院烟台海岸带研究所，山东 烟台 264003; 2. Key Laboratory of Coastal Environmental Processes and Ecological Restoration，Chinese Academy of Sciences（Yantai Institute of Coastal Research），Key Laboratory of Coastal Environmental Processes of Shandong Province，Yantai Institute of Coastal Research，Chinese Academy of Sciences，Yantai 264003，China

**Keywords:** 新污染物, 定量构效关系, 机器学习, 生物效应, emerging pollutants, quantitative structure-activity relationship（QSAR）, machine learning, biological effects

## Abstract

合成化合物在生产生活中被广泛使用，并不可避免地进入环境成为潜在的新污染物，进而与人类接触，危害人体健康。为了防治潜在新污染物的健康危害，需要全面评估已经和即将进入市场的化合物的毒性。基于实验的毒性评估速度远低于新化合物进入市场的速度，且传统毒性实验不仅耗费时间与经济成本，还会在不同实验室的实验结果之间产生争议，使毒性筛查标准不一。因此，亟需开发基于人工智能、机器学习标准的高通量毒性预测模型，以高效填补化合物毒性数据空缺。本研究基于机器学习方法对Tox21数据库中各种类别化合物进行毒性预测。化合物的结构数据使用简化分子线性输入规范（SMILES）格式表示，表征物理化学性质和实验条件的信息使用RDKit库和Mordred库编码为描述符。通过Python的Sklearn库与XGBoost库计算并筛选各变量的信息增益得到新的特征集，并依此建立毒性预测模型，实现对12类细胞核受体相关活性指标的精准预测。模型在12个数据集上的平均接收者操作特性曲线下面积（AUC）为0.84，所有训练和测试集数据均位于模型应用域内。外部验证结果表明，本研究所构建模型性能优于在Tox21挑战赛中的其他模型。通过SHAP算法对模型参数进行分析，解释了毒性机理，发现log *P*、分子拓扑结构、ZMIC、piPC等描述符是影响活性的主要原因。为了方便具有不同学科背景的研究人员和政策制定者使用该模型，将模型开发为可视化软件，允许以SMILES格式输入化合物结构并进行毒性预测。本研究开发的预测模型及其配套软件能够快速筛查新污染物的毒性，并为新化学品的安全设计提供指导。

## Abstract (en)

Emerging pollutants are substances that have recently been discovered or brought into focus， pose ecological or human-health risks， and have not yet been included in regulatory frameworks or for which existing management measures inadequately prevent and control their risks. Synthetic chemicals play key roles in progressing human society and improving quality of life. However， these chemicals may leak into the environment through unintentional or organized emissions during the life cycles of chemical-containing products， thereby becoming potential emerging pollutants and posing ecological and human-health threats. Many new chemicals are typically used without sufficient toxicity assessments； consequently， their potential threats are difficult to predict. Hence， effective toxicity assessments of existing and emerging chemicals are required to address this situation. Toxicity testing all chemicals is expected to be very time-consuming and economically expensive. In addition， there are discrepancies between experimental results from different laboratories leading to inconsistent toxicity-screening standards for emerging pollutants， which hinders preventing and controlling emerging pollutants and explaining their toxicity mechanisms. Addressing these issues requires the development of standard alternative toxicity-testing strategies that screen emerging pollutants in a high-throughput manner. In this study， machine-learning methods were used to predict the toxicities of various compounds in the Tox21 database. The RDKit and Mordred libraries were used to process structural data（presented in SMILES format） for compounds with the aim of generating molecular descriptors for their physicochemical properties. A set of refined features was screened through information-gain calculations and variable selection， and the data were fitted using Python’s Sklearn and XGBoost libraries. Prediction models were constructed based on the screened features using seven machine-learning algorithms in order to evaluate 12 different bioactive endpoints， including datasets related to endocrine disruption， DNA damage， and oxidative stress response， among others. Model performance was evaluated by calculating the accuracy of the test set， and data availability was characterized in terms of the application domain. All training and test data were found to be located in the application domain. The model was found to highly accurately predict 12 endpoints. This study clarified the relationship between the physicochemical properties of chemicals and nuclear receptor activity， and developed corresponding software tools. The model for the 12 Tox21 datasets exhibited an average area under the curve（AUC） of 0.84， and delivered better prediction performance than other participating models. Further insight into toxicological mechanisms was obtained through feature-importance analysis using Shapley Additive exPlanations（SHAPs）. The octanol-water partition coefficient（log *P*）， molecular topology， and ZMIC and piPC descriptors were identified as key parameters for predicting toxicity； these descriptors elucidate the relationship between chemical structure and biological interaction， thereby providing mechanistic explanations for compound toxicities. For example， high log *P* values are associated with high cell membrane permeability， which facilitates interactions between intracellular targets and endocrine receptors. The study also developed user-friendly quantitative structure-activity relationships（QSAR） prediction software. Designed for accessibility， this software enables researchers and policymakers to input compound structures in SMILES format and predict their toxicities without the need for specialized machine-learning expertise. The software automatically generates descriptors and predicts whether the input compounds are toxic or not. This study contributes to *in silico* methods that replace animal testing in future toxicity studies by integrating advanced machine-learning and interpretation methods. The predictive model and accompanying software enable the rapid screening of emerging pollutants and provide guidance for designing safer chemicals. These contributions are critical for advancing environmental safety and public health in the face of expanding chemical inventories.

化学品对于推动人类社会进步及提升民众生活质量起到了关键性的积极作用。然而，在含化学品的消费产品的整个生命周期内，这些化学品可能通过无组织排放、有组织排放等方式泄漏到环境中，成为潜在的新污染物^［1-4］^，对生态和人体健康构成威胁^［5］^。大量新化学品通常未经充分毒性评估即投入使用，导致其潜在威胁难以预测^［6］^。对化学品的环境风险进行预估和评估，能够有效支持化学品的风险管理与源头控制策略，对新污染物的风险防控至关重要。通过识别有害新污染物，限制其排放，可以降低其对环境和人体健康的危害^［7］^。

已有研究通过实验方法评估了新污染物的生态环境和人体健康危害^［8，9］^。然而，基于毒性实验的毒性评估效率较低，难以满足市场上大量新化学品所需的环境风险快速评估要求。截至2024年12月2日，美国化学文摘社（www.cas.org）登记的化合物已超过2.79亿种，并且还以每天1.5万余种的速度增加^［10］^。据统计，测定一个化合物毒性的平均时长达3.5年之久，全世界平均每年在毒理学实验中花费近30亿^［11］^。若要对所有化学品逐一进行毒性测试，将会耗费巨大的时间和经济成本。且不同实验室的实验结果之间存在争议，这使得新污染物毒性筛查标准不一，妨碍了新污染物防治和对其危害的机理解释。计算毒理学预测方法在新污染物筛查识别和风险评估中起着越来越重要的作用，开发传统实验技术的人工智能补充方法，能为色谱-质谱监测和化学品风险评估领域的人员提供有效参考^［12］^，进一步推动新污染物筛查及其交叉学科的基础或应用研究。例如，Miyawaki等^［13］^通过GC×GC-TOF-MS技术鉴定出未被收录于自动识别与定量系统（AIQS）数据库中的化合物，并利用计算毒理学模型对在河水中检测到的化合物以及AIQS数据库中的化合物进行毒性预测。依据预测的毒性值对这些化合物进行排序，从而确定优先级较高的污染物。研究结果表明，药品、个人护理品以及邻苯二甲酸二（2-乙基己基）酯在全年均被识别为河流中的高风险污染物^［13］^。

由于化合物与生物系统相互作用的机理复杂，以及化合物结构和性质的多样性，传统的统计学算法不足以建立高通量的新污染物毒性评估模型，人工智能和机器学习的快速发展为计算毒理学模型提供了新的解决思路^［14-16］^。Idakwo等^［17］^在Tox21挑战赛中以随机森林作为基础分类器，Bootstrap 聚合采样（bagging）作为继承策略，应用了包括随机森林、支持向量机等4种机器学习算法进行建模，4种算法的模型准确率（ACC）分布在0.58~0.79。该研究指出，毒物-靶标生物分子相互作用的特异性使许多毒性数据集非常不平衡，从而导致基于结构-活性关系的化学预测效果不佳；Moukheiber等^［18］^构建的模型预测了Tox21数据集中可能导致化合物毒性的蛋白质特征，表现最好的预测终点的接收者操作特性曲线（ROC）和平均ROC下面积（AUC）分别为0.92和0.90；Wu等^［19］^使用基于最小二乘支持向量机（LS-SVM）和随机森林算法组合的方法，对每个建模目标进行优化以平衡预测性和可解释性模型。尽管许多研究成功地构建了具有优秀预测能力的模型，但仍存在以下问题：（1）特征选择方法的应用不足，许多模型未对分子描述符的重要性进行筛选，导致模型复杂度增加且性能不稳定；（2）模型的应用域定义不够明确，导致预测结果的外推性较差；（3）未能充分利用多算法集成的优势。本研究针对以上问题，提出了基于特征筛选和多算法优化的高通量定量构效关系（QSAR）建模方法，并对模型的应用域进行了严格定义，开发更具泛化能力的标准毒性实验替代策略，为高通量筛查新污染物的毒性提供可能。

本研究基于Tox21数据库中的化合物，构建了包括化合物二维结构描述符和化合物活性的综合数据集；使用7种机器学习算法，构建了预测12种活性指标的计算毒理学模型；通过特征筛选方法，阐明了新污染物的结构与不同生物活性之间的关联，揭示了毒性机理；所构建的模型作为传统的污染物筛查和风险评估实验方法的补充工具，对化学品的高通量智能风险评估有一定参考价值。环境科学、色谱分析与机器学习的深度交叉，对实现新污染物的“识别-评估-管控”闭环管理具有重要实践价值。从数据集的获取到建模后毒性机理的解释，具体的研究框架及流程见图1。

**图1:**
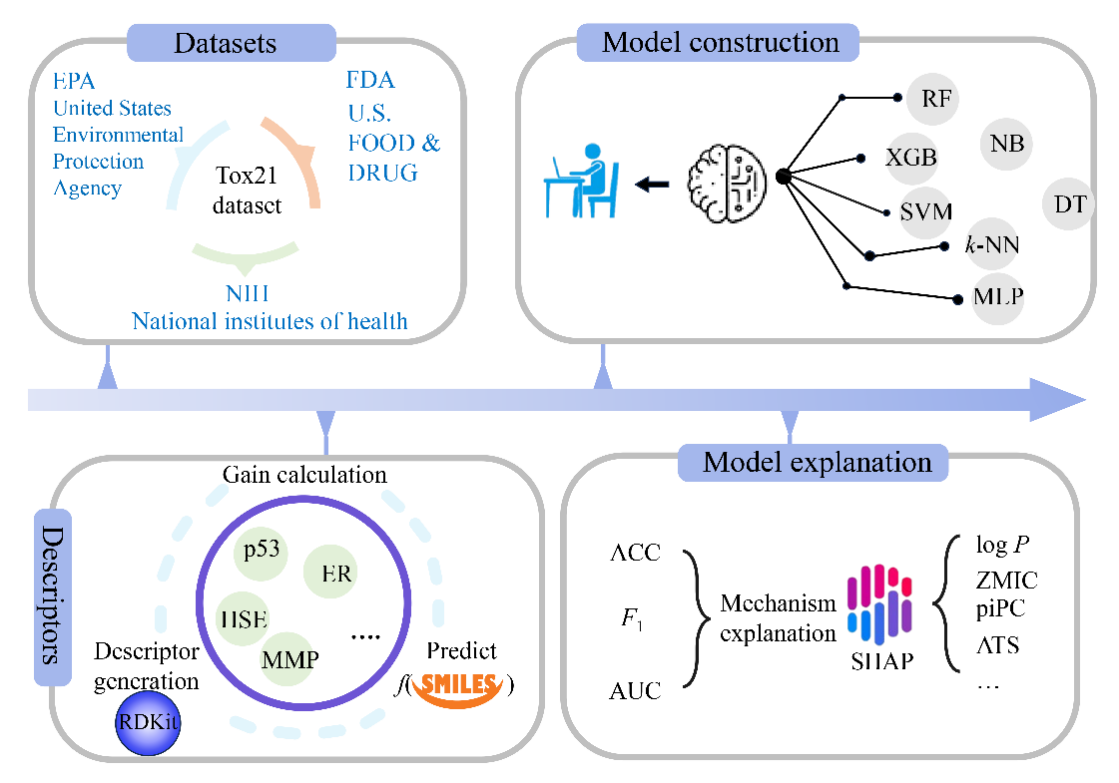
研究框架及流程图 RF： random forest； XGB： XGBoost； NB： naive bayes； DT： decision tree； MLP： multilayer perceptron； SVM： support vector machine； *k*-NN： *k*-nearest neighbors； ACC： accuracy； *F*_1_： *F*_1 _score； AUC： area under curve； p53： activation of p53 protein； HSE： agonism of heat shock response element； MMP： antagonism of metalloproteinase； ER： agonism of ERα.

## 1 实验与方法

### 1.1 数据来源

使用的化合物数据集来源于Tox21项目官网（https：//tox21.gov/data-and-tools/）。凭借其卓越的数据质量标准，该化合物数据集已在众多计算毒理学研究中得到广泛应用与验证^［19，20］^，具有较高的可信度和建模价值。数据集涵盖了12个不同的细胞核受体相关毒性预测终点。

（1）芳香烃受体（aryl hydrocarbon receptor，AhR）数据集涵盖了化合物与AhR相互作用的毒性数据。AhR是外源性物质（如二噁英等污染物）的传感器，其激活与肿瘤的产生和免疫系统紊乱等多种毒性效应相关。（2）雄激素受体（androgen receptor，AR）数据集中的化合物与内分泌干扰作用相关，AR在调控男性性征和生殖功能方面起着关键作用。（3）雄激素受体配体结合域（AR ligand binding domain，AR-LBD）数据集与AR数据集类似，该数据集描述了化合物与雄激素受体的配体结合域相互作用，主要表征化合物能否与AR受体结合，影响受体活性，从而改变信号传导过程。（4）抗氧化反应元件（antioxidant response element，ARE）数据集用于描述化合物能否激活抗氧化反应元件产生氧化应激反应。氧化应激是细胞面对外界毒性物质的防御机制之一。（5）芳香化酶（aromatase）数据集描述了化合物干扰内分泌的机制。Aromatase是一种单加氧酶，作用是催化甾体激素合成步骤中从雄激素到雌激素的芳香化反应，该反应是雌激素合成的关键步骤。（6）ATPase家族中AAA结构域5基因（*ATAD5*）与DNA修复相关。*ATAD5*数据集描述了化合物是否会影响与DNA损伤修复相关的通路，*ATAD5*的活性抑制可能导致DNA损伤积累，从而增加致癌风险。（7） 雌激素受体（estrogen receptor，ER）数据集评估化合物对雌激素受体的作用。雌激素受体在调控女性性征和生殖功能中起重要作用。（8） 雌激素受体配体结合域（ER ligand binding domain，ER-LBD）与ER类似，ER-LBD数据集描述了化合物与雌激素受体的配体结合域的相互作用。（9） 热休克元件（heat shock element，HSE）数据集描述了化合物是否能够激活热休克反应，该反应通常在细胞应对压力（如热应激或化学应激）时被激活，激活HSE通常与蛋白质损伤修复和保护细胞免受外界损伤有关。（10） 基质金属蛋白酶（matrix metalloproteinases，MMP）数据集评估化合物对基质金属蛋白酶的抑制或激活作用。MMPs是一类重要的蛋白水解酶，在细胞外基质的降解中发挥作用，与组织重塑、肿瘤侵袭和转移密切相关。（11） p53数据集描述了化合物是否会影响p53信号通路。p53蛋白是一种肿瘤抑制蛋白，负责检测DNA损伤并启动细胞修复或凋亡，在DNA损伤的背景下有助于识别可能致癌的化合物。（12） 过氧化物酶体增殖物激活受体（peroxisome proliferator-activated receptor gamma，PPARγ）数据集表征了化合物对PPARγ的激活或抑制作用。核受体PPARγ参与脂肪代谢和葡萄糖调节，与代谢相关疾病（如糖尿病）和脂肪生成相关。

### 1.2 分子描述符计算与模型的构建

本研究依据化合物化学文摘社（chemical abstracts service， CAS）编号和名称从Pubchem数据库中获取简化分子线性输入规范（SMILES）格式储存的化合物结构数据，利用Python中RDKit库和Mordred库计算了1 613个二维结构描述符，并删除了含有空值的描述符，最终数据包含780～1 385个描述符不等，其中包括拓扑指数、结构指数、二维原子对和环描述符等。

按照8∶2的比例将数据随机划分为训练集和验证集，利用7种机器学习算法（决策树（DT）、朴素贝叶斯（NB）、支持向量机（SVM）、*k*-近邻（*k*-NN）、随机森林（RF）、梯度提升（XGB）、多层感知机（MLP）和特征筛选方法）构建了模型。其中DT、NB、SVM、*k*-NN、MLP和RF算法通过Python中开源Sklearn库实现，XGB算法通过XGBoost库实现。RF是决策树的集成算法，通过构建多棵决策树进行投票，综合表决最终的预测结果，有效降低单一模型的过拟合风险；MLP是一种前馈神经网络，能够捕获学习非线性关系，适用于高维度数据集的学习，多种化学描述符数据集中存在非线性关系，使用MLP算法能够有效捕捉参数与结果之间的复杂非线性关联；*k*-NN算法基于实例数据的学习方法，通过计算样本之间的距离，实现分类，适合处理小数据集但对异常值敏感；XGB算法基于梯度提升决策树实现，具有随机森林算法的优点，在处理不平衡数据时有优势；DT算法通过递归地划分特征空间，来构建树状模型；NB算法是基于贝叶斯定理的分类算法，使用概率统计对样本数据分类，对缺失数据敏感度低；SVM算法按监督学习方式对数据进行二元分类，其决策边界是对学习样本求解的最大边距超平面，擅长特征维数较高的数据。特征筛选的具体过程：首先计算各变量的信息增益，并进行排序，根据特征重要性排名剔除排名最后一位的预测变量，得到一个新的特征集，并用新的特征集重新训练模型，以此类推，直到参与的变量剩下最后2个。特征筛选方法通过Python中开源Sklearn库实现。

### 1.3 模型的评价与验证

模型性能使用ACC和召回率的调和平均数（*F*_1_）总体预测，计算公式如下：


$$ACC=\frac{TP+TN}{TP+FP+TN+FN}$$ (1)

$$F_{1}=\frac{2TP\cdot SP}{SE+SP}$$ (2)

其中，TP是指正样本被正确识别的数量；FP是指误报的负样本数量；TN是指负样本被正确识别的数量；FN是指误报的正样本数量。

除了使用以上性能指标外，还通过计算ROC，计算了Tox21挑战赛中指定的验证集AUC，作为评估最终排名指标。AUC可以用来评价模型是否具有区分能力。使用AUC评价模型性能的一般规则：如果AUC≥0.8，则模型性能优秀；如果0.8>AUC≥0.7，则性能良好；如果0.7>AUC≥0.6，则性能一般；如果0.6>AUC≥0.5则性能较差。

### 1.4 模型应用域表征

在模型应用之前，需要定义其应用域，以确定模型的应用范围。由于各数据点之间的量纲不同，应用域评估会受到较大量纲的影响，因此在应用域评估之前需要对数据进行归一化处理，公式如下：


$$X_{nom}=\frac{X-X_{min}}{X_{max}-X_{min}}$$ (3)

其中，*X*_nom_指归一化后的数据，*X*指归一化前的数据，*X*_min_指数据最小值，*X*_max_指数据最大值。

本研究计算化合物参数之间的欧氏距离（ $D_{E}$ ）评价QSAR模型的应用域，公式如下：


$$D_{E}\left(x,\;\mu \right)=\sqrt[{}]{{\left(x-\mu \right)}^{T}(x-\mu )}$$ (4)

其中， ***x*** 为数据点的行向量，*μ*为 ***x*** 的均值。如果化合物与训练集中心点的距离超过训练集的最大距离，则认为该化合物在模型应用域之外。

## 2 结果与讨论

### 2.1 数据多样性

Tox21挑战赛的12个化合物数据集涵盖了多样化的化学物质和生物活性信息，为训练毒性预测模型提供了丰富的数据基础（其中AHR数据集1 901个数据点；AR数据集757个数据点；AR-LBD数据集605个数据点；Aromatase数据集713个数据点；ER数据集1 867个数据点；ER-LBD数据集883个数据点；PPARγ数据集443个数据点；ARE数据集2 189个数据点；*ATAD5*数据集675个数据点；HSE数据集851个数据点；MMP数据集2 247个数据点；p53数据集1 065个数据点）。每个数据集对应特定的毒性终点，如细胞凋亡、氧化应激反应、DNA损伤等，代表了不同的生物学过程。这些化合物数据集不仅包含药物和环境污染物，还包括广泛使用的工业化学品、农药等，反映了真实世界中化学品的多样性。

### 2.2 模型效果评价与对比

在12个数据集和使用的7个机器学习算法中，特征筛选后模型的AUC均≥特征筛选前模型的AUC（图2），表明特征筛选方法有利于提高模型的预测性能。此外，研究对比了不同算法在12个化合物数据集中的表现，发现RF、*k*-NN算法在AhR、AR、ER、PPARγ、ARE、*ATAD5*、HSE、p53和AR-LBD数据集中表现最佳，XGB、MLP算法在Aromatase、ER、ER-LBD和MMP数据集的表现最佳。

**图2:**
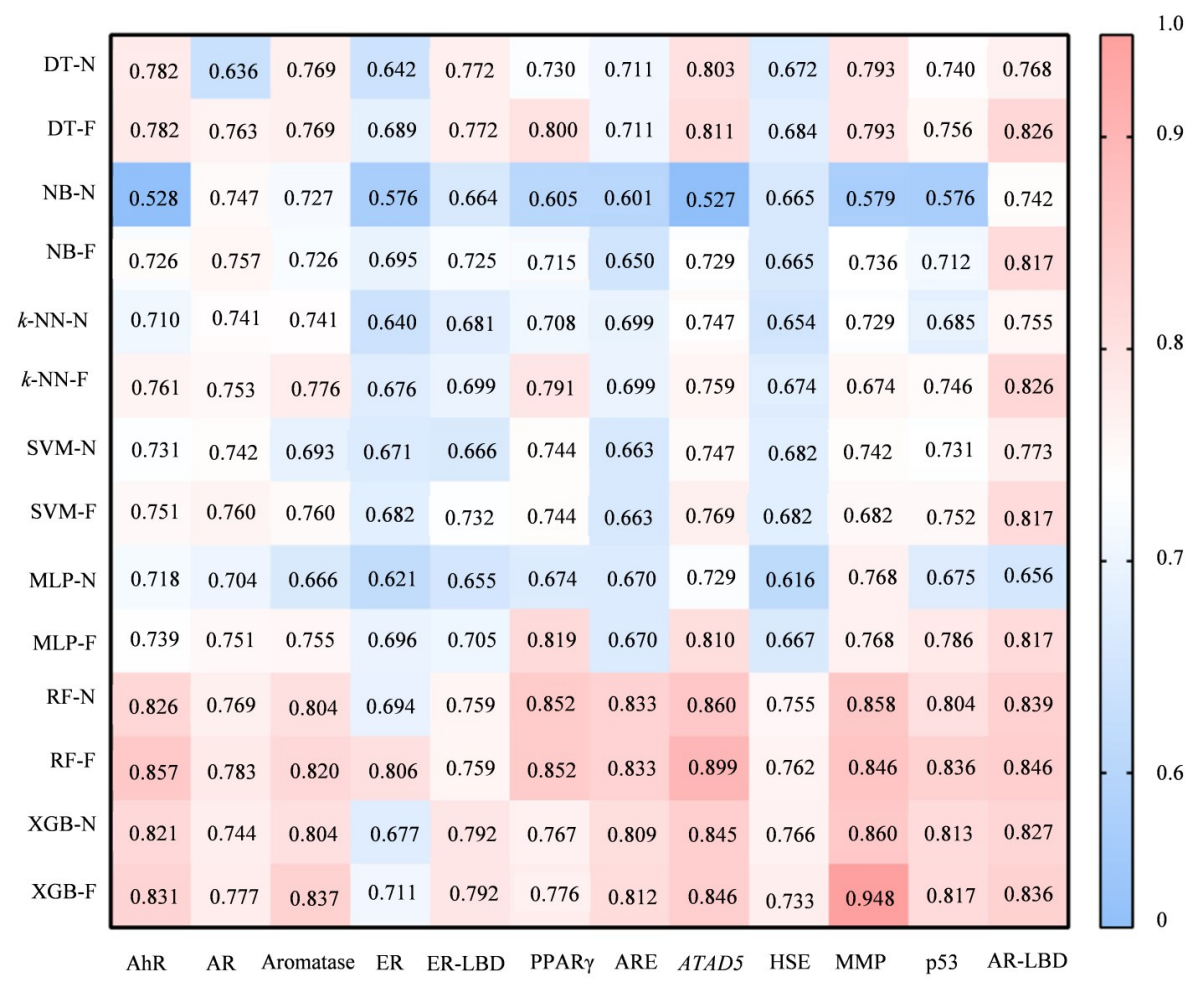
不同机器学习算法和特征筛选前后模型的AUC值 -N： before feature screening； -F： after feature screening； AhR： agonism of aromatase； AR： antagonism of nuclear receptor estrogen receptor α； Aromatase： inhibition of aromatase； ER-LBD： agonism of the ligand binding domain of ERα； PPARγ： agonism of peroxisome proliferator-activated receptor γ； ARE： agonism of antioxidant response element； *ATAD5*： antagonism of ATAD5 protein； AR-LBD： antagonism of the ligand binding domain of ERα.

使用RDKit库和Mordred库计算获得9种不同分子描述符（PubchemFingerprinter、Fingerprinter、MACCSFingerprinter、ExtendedFingerprinter、GraphOnlyFingerprinter、KlekotaRothFingerprinter、SubstructureFingerprinter、AtomPairs3DFingerprinter、EStateFingerprinter），并比较了以上9种描述符在XGB、RF、*k*-NN、MLP 4种机器学习算法中的性能表现（见图3）。综合来看，4种算法在ACC、*F*_1_、AUC均表现出均衡性，没有明显的短板，且在将所有数据集组合成All数据集，预测效果超过单独描述符组成的数据集，除*k*-NN外预测准确率均达0.85。

**图3:**
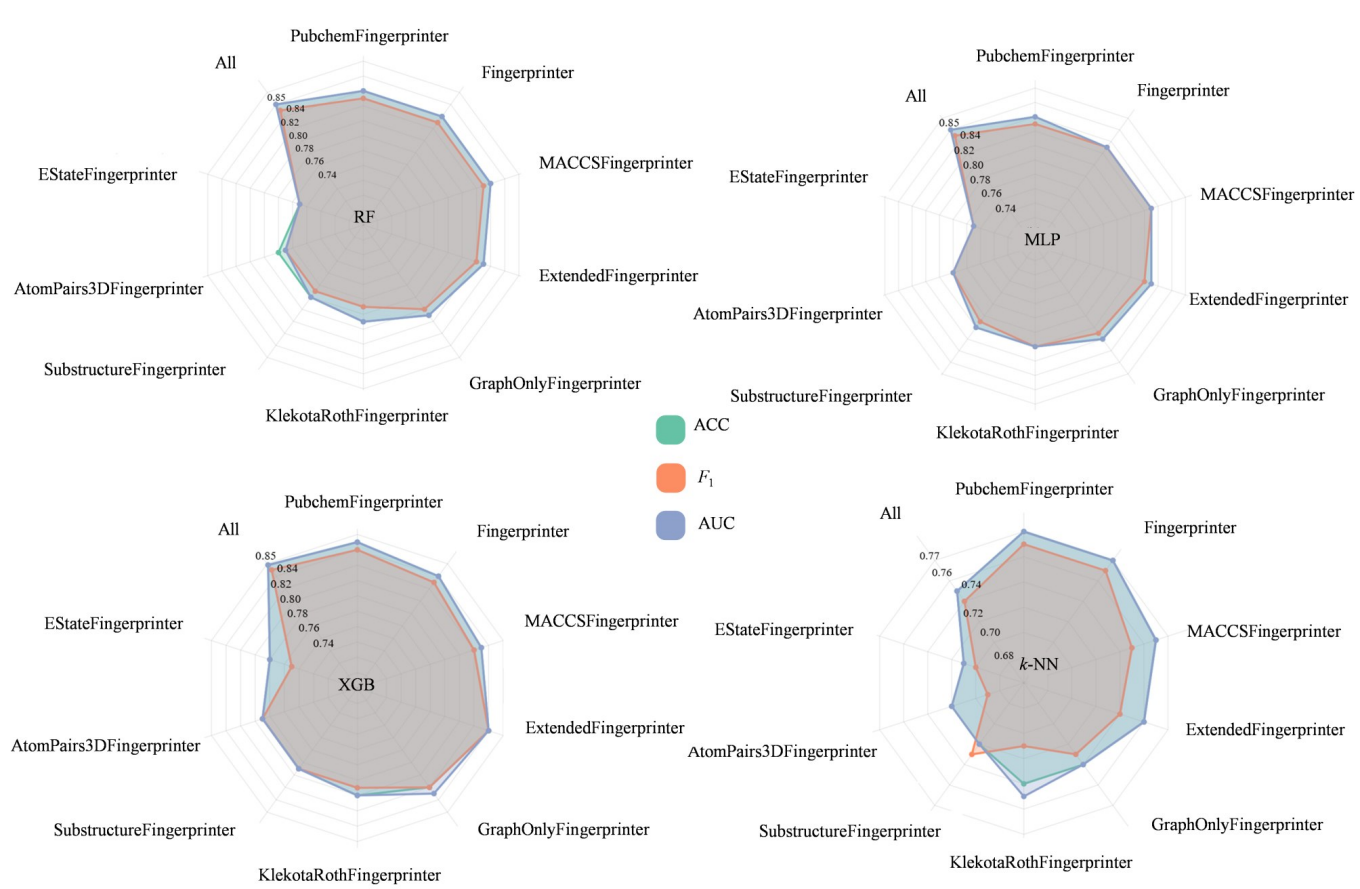
机器学习算法在9个描述符数据集的表现差异

对比不同算法在各个描述符数据集上的表现，发现每种算法在特定场景下的预测能力有所不同。RF、XGB、MLP算法在PubchemFingerprinter、Fingerprinter、MACCSFingerprinter、ExtendedFingerprinter、GraphOnlyFingerprinter描述符数据集的预测结果较好，测试集ACC、AUC、*F*_1_均大于0.8。XGB、RF、*k*-NN、MLP算法在KlekotaRothFingerprinter、SubstructureFingerprinter、AtomPairs3DFingerprinter、EStateFingerprinter描述符数据集上预测能力较强，测试集ACC、AUC、*F*_1_均大于0.7。

模型应用域表征结果说明，训练集和测试集中的所有化合物均位于模型的应用域中。数据集多样性以及模型应用域的表征进一步确保了模型具有广泛的适用性。此外，本研究利用构建的最优模型与Tox21各个参赛队模型结果进行了对比（表1）。结果表明，构建的模型在11个项目中的排名均未低于前5名，有5个是第1名，且平均AUC排名也是第1名。这充分证明本研究通过机器学习和特征筛选方法构建的QSAR模型具有良好的稳健性和预测能力。

**表 1:** 本研究构建模型的AUC值与Tox21 挑战赛其他队伍的对比

Team	Average	AhR	AR	AR-LBD	ARE	Aromatase	*ATAD5*	ER	ER-LBD	HSE	MMP	p53	PPARγ
This study	0.84^*^	0.86	**0.78**	**0.85**	0.83^*^	**0.84**	0.9^*^	0.81^*^	0.79	0.77	**0.95**	0.84	0.85^*^
AMAZIZ	**0.84**	0.91^*^	0.77	0.85	**0.81**	0.82	**0.83**	**0.81**	**0.81**	**0.84**	0.95^*^	0.84	0.83
dmlab	0.82	0.78	0.83^*^	0.82	0.77	0.84^*^	0.80	0.77	0.77	0.86^*^	0.95	0.88^*^	**0.83**
T	0.82	**0.91**	0.68	0.85^*^	0.80	0.83	0.81	0.78	0.80	0.81	0.94	**0.85**	0.82
microsomes	0.81	0.90	–	–	0.80	–	0.81	0.79	0.83^*^	–	–	0.83	0.72
filipsPL	0.80	0.89	0.74	0.74	0.76	0.78	–	0.77	–	0.77	0.93	0.82	–
Charite	0.79	0.90	0.69	0.79	0.74	0.78	0.75	0.71	0.80	0.85	0.88	0.83	0.70
RCC	0.77	0.87	0.76	0.75	0.76	0.79	0.67	0.78	0.76	0.76	0.92	0.80	0.64
frozenarm	0.77	0.87	0.74	0.72	0.70	0.74	0.73	0.75	0.79	0.75	0.86	0.80	0.8
ToxFit	0.76	0.86	0.74	0.76	0.70	0.74	0.73	0.73	0.75	0.69	0.86	0.80	0.79
CGL	0.76	0.87	0.74	0.57	0.75	0.75	0.73	0.76	0.73	0.78	0.88	0.82	0.74
SuperTox	0.74	0.85	–	0.56	0.71	0.74	–	–	–	–	0.86	0.73	–
kibutz	0.74	0.87	0.75	0.69	0.71	0.73	0.74	0.76	0.78	0.59	0.84	0.79	0.67
MML	0.73	0.87	0.69	0.66	0.70	0.71	0.75	0.75	0.71	0.65	0.85	0.82	0.65
NCI	0.72	0.81	0.63	0.59	0.78	0.70	0.71	0.48	0.70	0.86	0.85	0.75	0.74
VIF	0.71	0.83	0.80	0.61	0.64	0.67	0.66	0.73	0.74	0.72	0.80	0.65	0.67
Toxic	0.64	0.72	0.72	0.61	0.63	0.67	0.59	0.65	0.64	0.47	0.73	0.61	0.68
Swamidass	0.58	0.35	0.57	0.75	0.37	0.27	0.39	0.68	0.74	0.71	0.83	0.66	0.59

The best effect is bold text with “*”， and the second best effect is bold text.

### 2.3 模型机理解释

通过SHAP算法，分析化合物分子结构、物理化学性质与其生物活性之间的关系，揭示本研究所构建的模型中分子描述符与化合物毒性之间的作用规律，阐明毒性机理。

分子描述符反映了化合物的物理化学特性，这些特性与其在生物体内的行为密不可分。使用SHAP算法分析^［21，22］^筛选出化合物数据集中对结果影响最大的描述符（图4）。各描述符中英文对照及含义见表S1（https：//www.chrom-China.com）。表2为SHAP所计算的各描述符参数的影响力具体数值，据此所得正辛醇-水分配系数（log *P*）、分子极化率（ATS、AATS、ATSATSC、AATSC）、拓扑结构（Mic）描述符的自相关系数、信息内容描述符（ZMIC）以及分子的惯性矩（piPC）是影响模型预测结果的关键因素。这些描述符代表化合物的理化性质，其通过影响化合物的生物效应、代谢途径等影响化合物的细胞毒性。

**图4:**
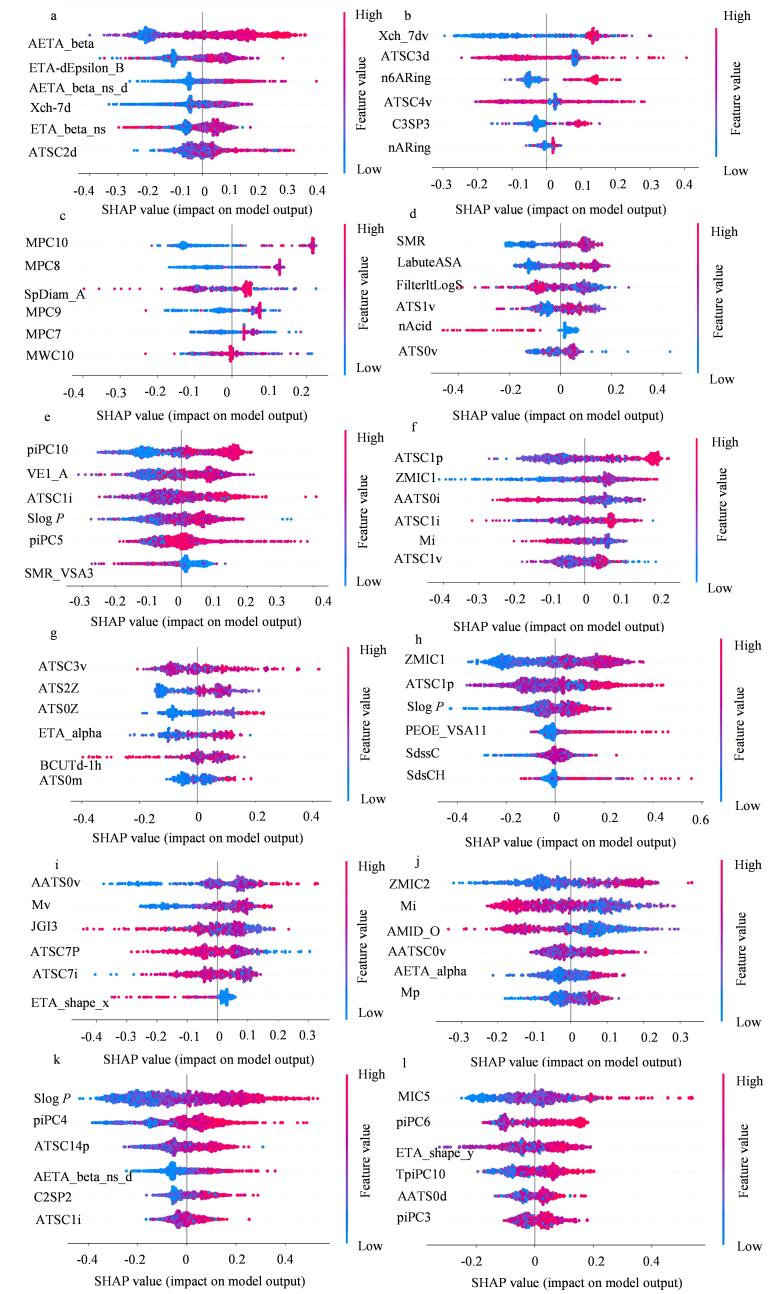
SHAP概要图 The descriptor data was subjected to SHAP analysis， and the top six descriptors with the greatest impact on the prediction results were screened out.

**表 2:** SHAP计算12个化合物数据集中各描述符参数的影响值

Dataset	Parameter	SHAP value/%	Dataset	Parameter	SHAP value/%
AhR	AETA_beta	30.6	PPARγ	ATSC3v	23.2
	ETA-dEpsilon_B	18.3		ATS2Z	21.3
	AETA_beta_ns_d	14.2		ATS0Z	15.0
	Xch-7d	13.4		ETA_alpha	14.0
	ETA_beta_ns	12.5		BCUTd-1h	13.8
	ATSC2d	11.0		ATS0m	12.6
AR	Xch_7dv	27.2	ARE	ZMIC1	24.9
	ATSC3d	19.4		ATSC1p	22.9
	n6ARing	19.2		SLogP	17.6
	ATSC4v	15.8		PEOE_VSA11	15.7
	C3SP3	9.8		SdssC	14.6
	nARing	8.5		SdsCH	4.2
AR-LBD	MPC10	33.0	*ATAD5*	AATS0v	22.4
	MPC8	16.8		Mv	18.2
	SpDiam_A	14.9		JGI3	17.5
	MPC9	13.4		ATSC7P	17.3
	MPC7	11.9		ATSC7i	14.7
	MWC10	10.3		ETA_shape_x	9.7
Aromatase	SMR	23.3	HSE	ZMIC2	25.0
	LabuteASA	22.1		Mi	19.3
	FilterltLogS	19.7		AMID_O	17.5
	ATS1v	12.2		AATSC0v	15.4
	nAcid	11.5		AETA_alpha	14.3
	ATS0v	11.1		Mp	8.6
ER	piPC10	23.5	MMP	SLogP	28.5
	VE1_A	21.1		piPC4	18.4
	ATSC1i	17.6		ATSC14p	15.9
	SLogP	14.7		AETA_beta_ns_d	14.9
	piPC5	12.8		C2SP2	12.8
	SMR_VSA3	10.2		ATSC1i	9.3
ER-LBD	ATSC1p	23.6	p53	MIC5	24.7
	ZMIC1	19.7		piPC6	21.1
	AATS0i	17.2		ETA_shape_y	16.5
	ATSC1i	14.9		TpiPC10	15.1
	Mi	13.3		AATS0d	12.4
	ATSC1v	11.5		piPC3	10.7

log *P*在预测化合物通过细胞膜的能力中发挥着重要作用。log *P*值越高，化合物越易于穿透磷脂双分子层，因此可能更容易与细胞内靶标相互作用^［23］^。在干扰内分泌干扰受体活性的预测中，亲脂性较高的化合物能够更有效地与核受体如ER和AR结合^［24］^，从而引发生物激素信号传导异常。

拓扑结构描述符的自相关系数参数通过计算捕捉分子中原子之间的距离、连接关系以及电荷分布信息来反映分子大小、形状等结构特征。这些特征影响分子与生物分子（如酶、受体、DNA）之间的相互作用。例如，结构上具有高度极性或带电区域的分子，可能更容易与生物大分子发生结合，引发不正常的生物反应，进而导致毒性效应^［25］^。

ZMIC描述符基于分子信息的复杂度和多样性计算所得，主要用于表征分子结构的信息熵和信息复杂度。较高的ZMIC值通常意味着分子结构更加复杂。在毒性机制中，ZMIC能够揭示化学物质的反应性和化学稳定性，高ZMIC值的分子通常具有更高的化学复杂性，这使得它们在与生物大分子（如蛋白质、DNA）接触时可能引发复杂的化学反应。例如，ZMIC可能通过产生有害的自由基引发DNA损伤或改变细胞代谢过程，从而导致细胞毒性^［26］^。ZMIC还可能与分子的跨膜能力和酶代谢相关。复杂的分子结构可能使其更难被生物体的代谢系统识别和处理，从而积累在体内，对目标组织造成慢性损害。

piPC描述符基于分子在三维空间中的惯性矩计算，反映了分子如何在三维空间内结构与旋转。在毒性机制方面，piPC描述符揭示了分子的三维结构和对称性。分子对称性较高的化学物质通常更稳定，意味着它们可能具有较低的反应性，从而减少对生物体的毒性。然而，不规则或较为复杂的分子结构可能导致分子在与生物大分子结合时产生较大的空间适配性，从而增强其与受体或酶的结合能力，引发不良的生物反应。此外，piPC能够揭示分子的机械稳定性，影响其在体内的生物降解性和蓄积情况。不稳定的物质可能会在体内产生有害的代谢产物，导致细胞损伤或慢性毒性反应。例如，分子中某些结构单元的旋转或扭曲可能使其更容易与细胞膜或受体发生异常反应，从而引发毒性效应^［27］^。

分子描述符是理解化合物毒性机制的关键工具。通过分析计算分子描述符与毒性终点之间的联系，可以更好地预测化合物在不同生物系统中的毒性表现。在Tox21化合物数据集中，对多个毒性终点的分析进一步揭示了多种分子描述符在不同生物学背景下的作用机制。这种寻找分子描述符与导致毒性机理的方式，为新污染物毒性预测提供了科学的依据，同时也为设计更加安全的化学品和降低环境风险提供了参考价值。

此外，针对不同毒性终点选择合适的算法和分子描述符组合也是提升建模性能的关键。例如，对于AhR和ARE终点，使用RF结合指纹特征可以获得更好的ACC；对于Aromatase和ER终点，使用XGB结合拓扑和电性质相关描述符可以获得更好的预测性能。

### 2.4 软件工具及功能概述

以QSAR模型为代表的计算毒理学技术可实现化学品危害性的高通量预测，填补相关数据缺失。近年来，计算毒理学工具也获得了一些监管部门的认可^［28］^。虽然目前已经开发了一批高性能机器学习模型，但它们在纳米安全管理中的应用仍受到极大限制。这是由于机器学习模型（特别是黑箱、灰箱模型）在实际应用中存在明显的局限性：对于具有不同学术背景的研究人员和决策制定者，难以快速运用机器学习相关知识运行其他研究人员开发的模型。为了最大限度方便研究人员和决策制定者利用计算模拟的方法快速便捷地评估潜在新污染物的危害性，本研究基于所构建的机器学习模型开发了软件工具QSAR毒性预测软件（1.0.0），以便所有人可以轻松地执行机器学习预测程序，而不需要提前掌握专门的机器学习知识。QSAR毒性预测软件可在https：//github.com/bingbushangshu/Tox21下载，在Windows10操作系统上运行，将化合物SMILES信息输入软件，选择目标毒性预测模型即可进行毒性预测（图5）。软件能自动识别错误的SMILES格式，并输出化合物的二维结构图与毒性预测结果。毒性预测结果可用于新污染物的危害性评估和新化学品的绿色安全设计。

**图5:**
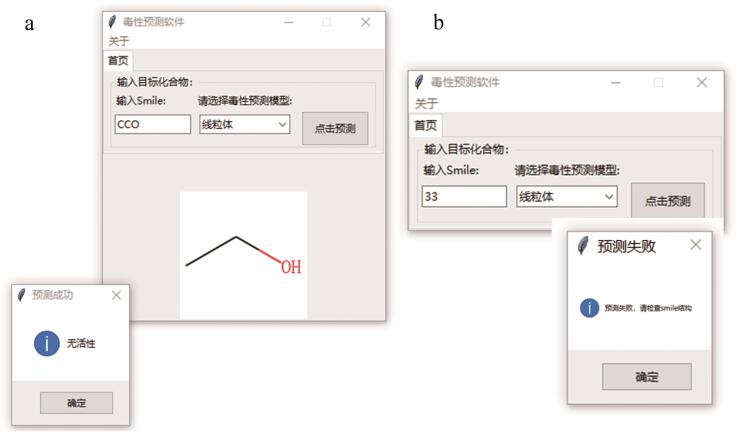
QSAR预测软件毒性预测功能界面 a. prediction success interface； b. prediction failure interface.

## 3 结论

本研究建立了涵盖12种参数共14 196个数据点的多维数据集，用于模型训练。采用多种机器学习算法，结合信息增益排序和特征重要性分析，构建了新污染物生物效应高通量预测模型集群，并定义了模型的应用域。基于SHAP模型解释算法能够帮助揭示新污染物的毒性机理。本研究所构建的计算毒理学模型及其软件作为传统的污染物毒性实验方法的补充工具，对化学品的风险评估有一定的参考价值。对于非靶向筛查技术识别出的缺乏毒性信息的新污染物，进行模拟风险评估，有利于筛选出高风险化合物，为新污染物管控措施的制定提供依据。
